# Influence of chemical denaturants on the activity, fold and zinc status of anthrax lethal factor

**DOI:** 10.1016/j.bbrep.2015.03.004

**Published:** 2015-03-24

**Authors:** Suet Y. Lo, Crystal E. Säbel, Jonathan P.J. Mapletoft, Stefan Siemann

**Affiliations:** a Department of Chemistry and Biochemistry, Laurentian University, Sudbury, Ontario, Canada; b Bharti School of Engineering, Laurentian University, Sudbury, Ontario, Canada

**Keywords:** CD, circular dichroism, cps, counts per second, DPA, dipicolinic acid, EDTA, ethylenediaminetetraacetic acid, EF, edema factor, LF, anthrax lethal factor, MWCO, molecular weight cut-off, PA, protective antigen, PAR, 4-(2-pyridylazo)resorcinol, *S*-*p*NA, lethal factor substrate, SASA, solvent-accessible surface area, SOD, superoxide dismutase, ZnLF, zinc-containing lethal factor, Protein folding, Zinc, 4-(2-pyridylazo)resorcinol, Lethal factor, Tryptophan fluorescence, Chemical denaturants

## Abstract

Anthrax lethal factor (LF) is a zinc-dependent endopeptidase which, through a process facilitated by protective antigen, translocates to the host cell cytosol in a partially unfolded state. In the current report, the influence of urea and guanidine hydrochloride (GdnHCl) on LF׳s catalytic function, fold and metal binding was assessed at neutral pH. Both urea and GdnHCl were found to inhibit LF prior to the onset of unfolding, with the inhibition by the latter denaturant being a consequence of its ionic strength. With the exception of demetallated LF (apoLF) in urea, unfolding, as monitored by tryptophan fluorescence spectroscopy, was found to follow a two-state (native to unfolded) mechanism. Analysis of the metal status of LF with 4-(2-pyridylazoresorcinol) (PAR) following urea or GdnHCl exposure suggests the enzyme to be capable of maintaining its metal ion passed the observed unfolding transition in a chelator-inaccessible form. Although an increase in the concentration of the denaturants eventually allowed the chelator access to the protein׳s zinc ion, such process is not correlated with the release of the metal ion. Indeed, significant dissociation of the zinc ion from LF was not observed even at 6 M urea, and only high concentrations of GdnHCl (>3 M) were capable of inducing the release of the metal ion from the protein. Hence, the current study demonstrates not only the propensity of LF to tightly bind its zinc ion beyond the spectroscopically determined unfolding transition, but also the utility of PAR as a structural probe.

## Highlights

• Lethal factor (LF) is strongly inhibited by guanidine hydrochloride.
• Except of apoLF in urea, unfolding follows a two-state mechanism.
• LF shields and retains its zinc ion in an unfolded state.
• Pyridylazoresorcinol is a useful probe to assess metal accessibility and release.

## Graphical abstract

**Figure:**
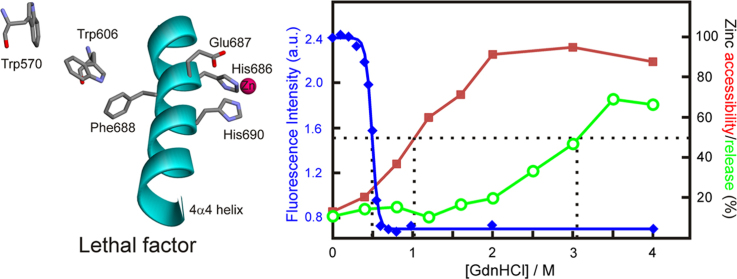

## 1 Introduction

Anthrax lethal factor (LF) is a zinc-dependent metalloendopeptidase and, along with protective antigen (PA) and edema factor (EF), one of the three protein components constituting the anthrax toxin [1], [2], [3]. While PA is quintessential for the delivery of LF and EF to the host cell cytosol *via* the formation of a heptameric or octameric pore in the membrane of the acidified endosomal compartment [4], [5], [6], [7], EF has been shown to be a calcium and calmodulin-dependent adenylyl cyclase involved in the dysregulation of water homeostasis by catalyzing the production of excessive amounts of cyclic AMP [8].

LF belongs to the family of gluzincins and exerts its deleterious intracellular action by removing N-terminal segments from most members of the mitogen-activated protein kinase kinase (MAPKK) family of signaling proteins [9], [10], and from NOD-like receptor protein 1 (Nlrp1) resulting in the activation of the inflammasome and macrophage death [11], [12], [13]. The Zn^2+^ ion in the active site of LF is bound tetrahedrally to the side chains of His686, His690 and Glu735, and to the nucleophilic water molecule responsible for the cleavage of the scissile peptide bond in the substrates [14]. In addition, Glu687, which is part of the thermolysin-like HExxH consensus motif [15], functions as the general base in the catalytic mechanism of LF [14].

As outlined above, PA (in its oligomeric, pore-forming state) mediates the translocation of LF and EF from the endosome to the cytosol. In view of the constriction (to ~15 Å) of the central lumen of the pore, it appears unlikely that secondary structural motifs larger than a single *α*-helix are capable of migrating through the pore [16], [17]. Hence, the translocation of LF and EF through the PA channel necessitates the partial, pH-assisted unfolding of these enzymatic entities [17], [18], [19]. Whether LF׳s Zn^2+^ ion remains associated to the protein during PA-mediated translocation is currently unknown. While it has recently been shown that acidification of LF to pH 5 (pH conditions encountered in late endosomes) leads to partial (~30%) demetallation of the enzyme active site [20], the effect of unfolding on the integrity of the protein׳s Zn^2+^-binding motif has not been investigated previously.

The current studies were designed to elucidate the effect of ionic and non-ionic denaturants (guanidine hydrochloride and urea, respectively) on the catalytic competence and fold of full-length LF, as well as their influence on the binding of the active site Zn^2+^ ion at neutral pH. Structural perturbations as a consequence of denaturant exposure were investigated by intrinsic tryptophan fluorescence spectroscopy, and revealed the protein to typically unfold *via* a two-state (native to unfolded) mechanism. Furthermore, the analysis of the metal status of LF following supplementation of the protein with urea/GdnHCl at moderate concentrations suggests the enzyme to be capable of retaining its metal ion passed the spectroscopically observed unfolding transition in a chelator-inaccessible form. The actual dissociation of the Zn^2+^ ion from the protein appears to require much higher concentrations of the chaotropes, hence, highlighting LF׳s propensity to tightly bind its metal ion even when in a denatured state.

## 2 Materials and methods

### 2.1 General

Chromogenic anthrax lethal factor protease substrate II, *S*-*p*NA (Ac-GYβARRRRRRRRVLR-*p*NA, *p*NA=*para*-nitroanilide) was obtained from Biomatik (Cambridge, ON, Canada). Urea, guanidine hydrochloride (GdnHCl) and guanidine isothiocyanate (GdnSCN) were purchased from BioShop (Burlington, ON, Canada), and were of the highest available purity. All other chemicals were purchased from Sigma-Aldrich (St. Louis, MO). All solutions were prepared using MilliQ ultrapure water (≥18.2 MΩ cm resistivity).

### 2.2 Isolation of LF

Wild-type LF (ZnLF) was expressed in *Bacillus megaterium* and purified according to published procedures [21]. The Zn^2+^ content of LF was assessed using the chromophoric chelator 4-(2-pyridylazo)resorcinol (PAR) as outlined previously [22], and was found to be 0.95 (±0.10) Zn^2+^/protein molecule. ApoLF with a residual Zn^2+^ content of less than 0.03 Zn^2+^/LF molecule was obtained by demetallation of ZnLF with the aid of dipicolinic acid (DPA) and ethylenediaminetetraacetic acid (EDTA) as documented recently [23].

### 2.3 Enzymatic assays

The enzymatic activity of LF preparations was assessed using the chromogenic *S*-*p*NA substrate according to published procedures [22], [24]. In a typical assay (final volume: 0.1 mL), LF (50 nM) in Hepes buffer (50 mM, pH 7.4) as allowed to equilibrate at room temperature for 1 min prior to the initiation of the reaction by the addition of substrate (final concentration of 10 μM). The progress of substrate hydrolysis was monitored at 405 nm using a Cary 60 UV–vis spectrophotometer (Agilent, Santa Clara, CA). The half-inhibitory concentrations (IC_50_ values) of MgSO_4_, Gdn-containing salts as well as their sodium analogs were determined in a fashion analogous to that outlined above, except for the incubation of the enzyme in the presence of these salts for 30 min prior to the supplementation of the assay with *S*-*p*NA.

### 2.4 Accessibility of Zn^2+^ to chelation by PAR

The accessibility of LF-bound Zn^2+^ to chelation by PAR as a function of denaturant concentration was assessed in a 96-well microplate format. In a volume of 190 μL (per well), ZnLF was exposed to GdnHCl or urea at various concentrations in Hepes buffer (50 mM, pH 7.4) for 1 h and 24 h at room temperature prior to the addition of 10 μL PAR (1 mM) to yield final concentrations of 5 μM and 50 μM with respect to the enzyme and chelator, respectively. Following supplementation with PAR, the progress of the reaction of the chelator with LF׳s Zn^2+^ ion was monitored spectrophotometrically at 500 nm for 60 min (10 s intervals) using a BioTek Epoch microplate spectrophotometer (Winooski, VT). Standards containing ZnSO_4_ (0–6 μM), denaturant (at concentrations identical to those of the LF samples) and PAR (50 μM) were prepared and measured under analogous conditions alongside the protein samples. The degree of complexation of LF׳s Zn^2+^ ion by PAR was determined based on the linear relationship between the concentration of Zn^2+^ in the standard and the absorbance at 500 nm (assessed after each 10 s time interval).

### 2.5 Metal release studies

The release of Zn^2+^ from LF as a function of the denaturant concentration was determined with the aid of PAR as follows: In a final volume of 550 μL, LF (5 μM) in Hepes buffer (50 mM, pH 7.4) was exposed to urea or GdnHCl at the desired concentration for 1 h at room temperature. Following incubation, a 275 μL aliquot was withdrawn from the mixture and immediately subjected to filtration using a 0.5 mL Amicon Ultra-15 centrifugal filter (30 kDa MWCO; Millipore, Bedford, MA). The remaining sample (275 μL) was incubated for another 23 h prior to Amicon filtration. The concentration of released Zn^2+^ was assessed spectrophotometrically by incubating 190 μL of each filtrate with 10 μL of PAR (1 mM) for 1 h, followed by monitoring of the absorbance of the PAR-Zn^2+^ complex at 500 nm. Standards containing ZnSO_4_ (0–6 μM), denaturant (at the appropriate concentration) and PAR (50 μM) were measured under analogous conditions, and served as the basis for the determination of the amount of Zn^2+^ released from LF.

### 2.6 Intrinsic tryptophan fluorescence spectroscopy and data analysis

In a total volume of 0.8 mL, LF at a final concentration of 0.5 μM in Hepes buffer (50 mM, pH 7.4) was incubated at room temperature in the absence and presence of GdnHCl or urea (0–6 M) for 1 h or 24 h prior to monitoring protein fluorescence. Samples of apoLF were prepared and measured analogously except for the inclusion of dipicolinic acid at a final concentration of 0.1 mM to prevent partial reconstitution by trace amounts of Zn^2+^ present in the medium. Samples of Zn^2+^-reconstituted apoLF were prepared by exposing the apoprotein (2 μM) to Zn^2+^ (5 μM) for 1 h prior to diluting the sample with Hepes buffer containing the desired amount of denaturant to achieve final concentrations of 0.5 μM and 1.25 μM with respect to the protein and metal ion, respectively. It is important to note that Zn^2+^-reconstituted apoLF obtained in this manner was found to regain its full catalytic activity.

Steady-state fluorescence emission spectra of ZnLF, apoLF and Zn^2+^-reconstituted apoLF were recorded at 20 °C using an OLIS RSM1000 spectrofluorometer (Bogart, GA) equipped with a 150 W Xenon arc lamp, a photon counter and a Julabo CF31 water bath (Allentown, PA) for temperature control. The excitation wavelength was set to 295 nm with a 5 nm bandpass. Emission spectra were obtained from 300 to 400 nm in 0.5 nm increments using an integration time of 1 s, and a bandpass of 4 nm. All spectra were processed by subtraction of spectra obtained for the corresponding denaturant/buffer samples in the absence of protein, followed by smoothing using the OLIS GlobalWorks software.

The degree of LF unfolding was assessed on the basis of the fluorescence intensities at 333 nm (FI_333_), the wavelength at which maximal emission is observed for the native state of the protein. The FI_333_ values were determined from the recorded emission spectra, and their dependence on the concentration of denaturant was plotted and fit by least-squares non-linear regression to a linear extrapolation model [25], [26] represented by Eq. (1) [27], [28], describing a two-state mechanism (native to unfolded, N→U), and/or by Eq. (2) [28], describing a three-state mechanism (native, intermediate, unfolded),

(1) $${\mathrm{FI}}_{obs}=\frac{{\mathrm{FI}}_{N}+{\mathrm{FI}}_{U}\times \exp\left(-\frac{\Delta G_{N\to U}^{0}-m_{N\to U}c}{\mathrm{RT}}\right)}{1+\exp\left(-\frac{\Delta G_{N\to U}^{0}-m_{N\to U}c}{\mathrm{RT}}\right)}$$

(2) $${\mathrm{FI}}_{obs}=\frac{{\mathrm{FI}}_{N}+{\mathrm{FI}}_{I}\times \exp\left(-\frac{\Delta G_{N\to I}^{0}-m_{N\to I}c}{\mathrm{RT}}\right)+{\mathrm{FI}}_{U}\times \exp\left(-\frac{\Delta G_{N\to I}^{0}-m_{N\to I}c}{\mathrm{RT}}\right)\times \exp\left(-\frac{\Delta G_{I\to U}^{0}-m_{I\to U}c}{\mathrm{RT}}\right)}{1+\exp\left(-\frac{\Delta G_{N\to I}^{0}-m_{N\to I}c}{\mathrm{RT}}\right)+\exp\left(-\frac{\Delta G_{N\to I}^{0}-m_{N\to I}c}{\mathrm{RT}}\right)\times \exp\left(-\frac{\Delta G_{I\to U}^{0}-m_{I\to U}c}{\mathrm{RT}}\right)}$$

where ${\mathrm{FI}}_{obs}$ is the observed fluorescence intensity at 333 nm, FI_*N*_, FI_*I*_, and FI_*U*_ represent the intensities of the native, intermediate and unfolded states, $\Delta G^{0}$ denotes the Gibbs free energy of a transition in the absence of denaturant, *m* is the denaturant dependence of $\Delta G^{0}$, *c* is the concentration of denaturant, *R* is the gas constant (1.987 cal K^−1^ mol^−1^), and *T* is the temperature (*i.e.*, 293.15 K). All data fitting was performed with the aid of Grafit 4.0 (Erithacus Software Ltd., Staines, UK) with $\Delta G^{0}$, *m* and FI_*I*_ (for the three-state model) serving as the fitting parameters.

The midpoint concentrations (*C*^*mid*^) for the native to unfolded transition (two-state mechanism), and native to intermediate as well as intermediate to unfolded transitions (three-state mechanism) were calculated from the fitting parameters as shown in Eq. (3).

(3) $$C_{N\to I}^{mid}=\;\Delta G_{N\to I}^{0}/m_{N\to I};\;\;C_{I\to U}^{mid}=\Delta G_{I\to U}^{0}/m_{I\to U};\;\;C_{N\to U}^{mid}=\;\Delta G_{N\to U}^{0}/m_{N\to U}.$$

## 3 Results

### 3.1 Effect of denaturants and salts on LF activity

As shown in Table 1, GdnHCl was found to inhibit LF with an IC_50_ value of 0.14 M. To assess whether the inhibition of the enzyme by GdnHCl is a consequence of its chaotropic nature, the ionic strength of the medium or a combination of both, the effect of two other Gdn-containing salts (GdnSCN and (Gdn)_2_SO_4_) and their corresponding sodium analogs on the catalytic function of LF was investigated. While the strong chaotrope GdnSCN displayed an inhibitory potency identical to that of GdnHCl, the non-chaotrope (Gdn)_2_SO_4_ exhibited a threefold lower IC_50_ value, suggesting that LF inhibition is not correlated to the propensity of these salts to unfold proteins (see Table 1). Instead, the data is consistent with the impairment of LF function being a result of ionic strength since at their respective IC_50_ concentrations, all three Gdn salts had essentially identical ionic strengths. In agreement with this observation, studies on the sodium analogs and MgSO_4_ indicate LF inhibition to be independent of the nature of the chemical constituents of these salts (be they Gdn or sodium, chloride or sulfate), but rather to be directly correlated to the ionic strength of the medium with the half-inhibitory concentration being observed at an ionic strength of ~0.17 M (with the contribution by the buffer taken into account). The slightly higher inhibitory potency of MgSO_4_ (as evidenced by the lower ionic strength at the IC_50_ value) can be attributed to Mg^2+^ since this metal ion has previously been shown to inhibit LF at millimolar concentrations [22]. Furthermore, the non-ionic chaotrope urea was found to inhibit LF at much higher concentrations (IC_50_=1.05±0.10 M), further supporting the notion of ionic strength being the main contributor to the inhibition of LF by GdnHCl.

**Table 1:** Effect of salts and urea on LF activity.

	Relative ionic strength	IC_50_ (mM)	Ionic strength at IC_50_ (mM)^a^
GdnHCl	1	140 (±10)	165
GdnSCN	1	140 (±12)	165
(Gdn)_2_SO_4_	3	50 (±5)	175
NaCl	1	150 (±15)	175
NaSCN	1	150 (±10)	175
Na_2_SO_4_	3	50 (±7)	175
MgSO_4_	4	25 (±4)	125
Urea	0	1050 (±100)	25

LF (50 nM) in Hepes buffer (50 mM, pH 7.4) was incubated in the presence of salts or urea for 30 min prior to the initiation of the assay by addition of *S*-*p*NA (10 μM). IC_50_ values represent the mean (±1 s.d.) of three independent experiments.

a The ionic strength of 50 mM Hepes at pH 7.4 (*i.e.*, 25 mM) was taken into account in the calculation of the ionic strength at the IC_50_ value.

### 3.2 Unfolding of LF

**Fig. 1:**
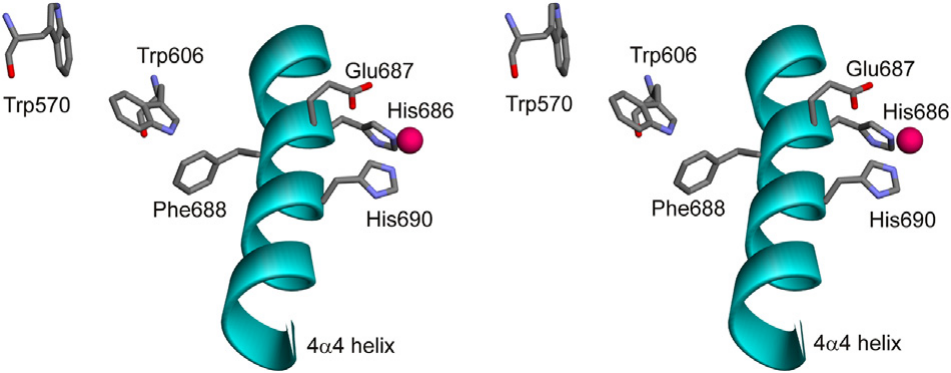
Stereoview of the spatial relationship between the active site of LF and vicinal tryptophan residues. The active site Zn^2+^ ion is depicted in magenta, whereas the 4α4 helix harboring the amino acid residues of the HExxH motif are shown in cyan. For the sake of clarity, the 4α7 helix containing the Zn^2+^-coordinating Glu735 residue has been omitted. The image was generated with Discovery Studio 3.5 (Accelrys, San Diego, CA) using coordinates deposited under the pdb entry 1J7N [14].

LF possesses five tryptophan residues located in the C-terminal domain (domains 2–4) of the protein (see Fig. S1). While three Trp residues are positioned in the domain adjacent to the N-terminal, PA-binding domain of LF (Trp 271, Trp 281, Trp 501), the remaining two residues (Trp570, Trp606) are buried in the vicinity of the active site of the enzyme. Furthermore, the aromatic moieties of Trp570, Trp606 and Phe688 interact with each other in an edge-to-face fashion (see Fig. 1). Phe688 is part of the prototypical, thermolysin-like HExxH motif found in many zinc hydrolases, and is located on the 4α4 helix, which harbors the two histidine residues (His686 and His690) involved in Zn^2+^ coordination, as well as Glu687 serving as the general base in the catalytic mechanism of the enzyme [14]. Hence, it is not unreasonable to assume that denaturant-induced structural perturbations of LF׳s active site, and potential labilization of its metal ion correlate with changes in the environment surrounding Trp570 and Trp606, a feature observable by intrinsic tryptophan fluorescence spectroscopy.

**Fig. 2:**
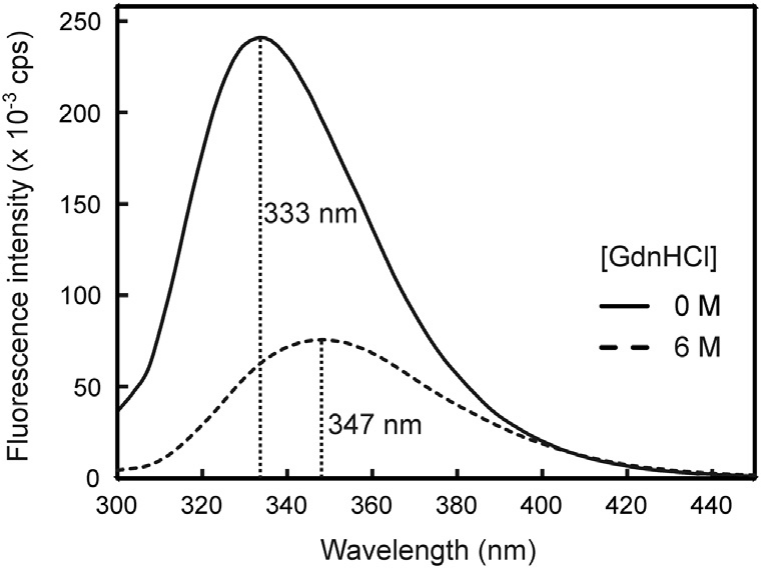
Tryptophan fluorescence spectra of LF in the absence and presence of 6 M GdnHCl. LF (0.5 µM) was incubated in the absence (solid line), and presence of 6 M GdnHCl (dashed line) for 24 h at room temperature prior to recording emission spectra. The excitation wavelength was set to 295 nm to allow for the selective excitation of LF׳s tryptophan residues.

As shown in Fig. 2, the denaturation of LF is accompanied by a redshift in the tryptophan fluorescence emission spectrum as evidenced by the change of the wavelength of maximum emission (*λ*_*max*_) from 333 nm to 347 nm, and by a 3.8-fold decrease in fluorescence intensity (at 333 nm). It is pertinent to note that the transition between the native and denatured states is reversible as evidenced by the return of the emission maximum to 333 nm (data not shown).

**Fig. 3:**
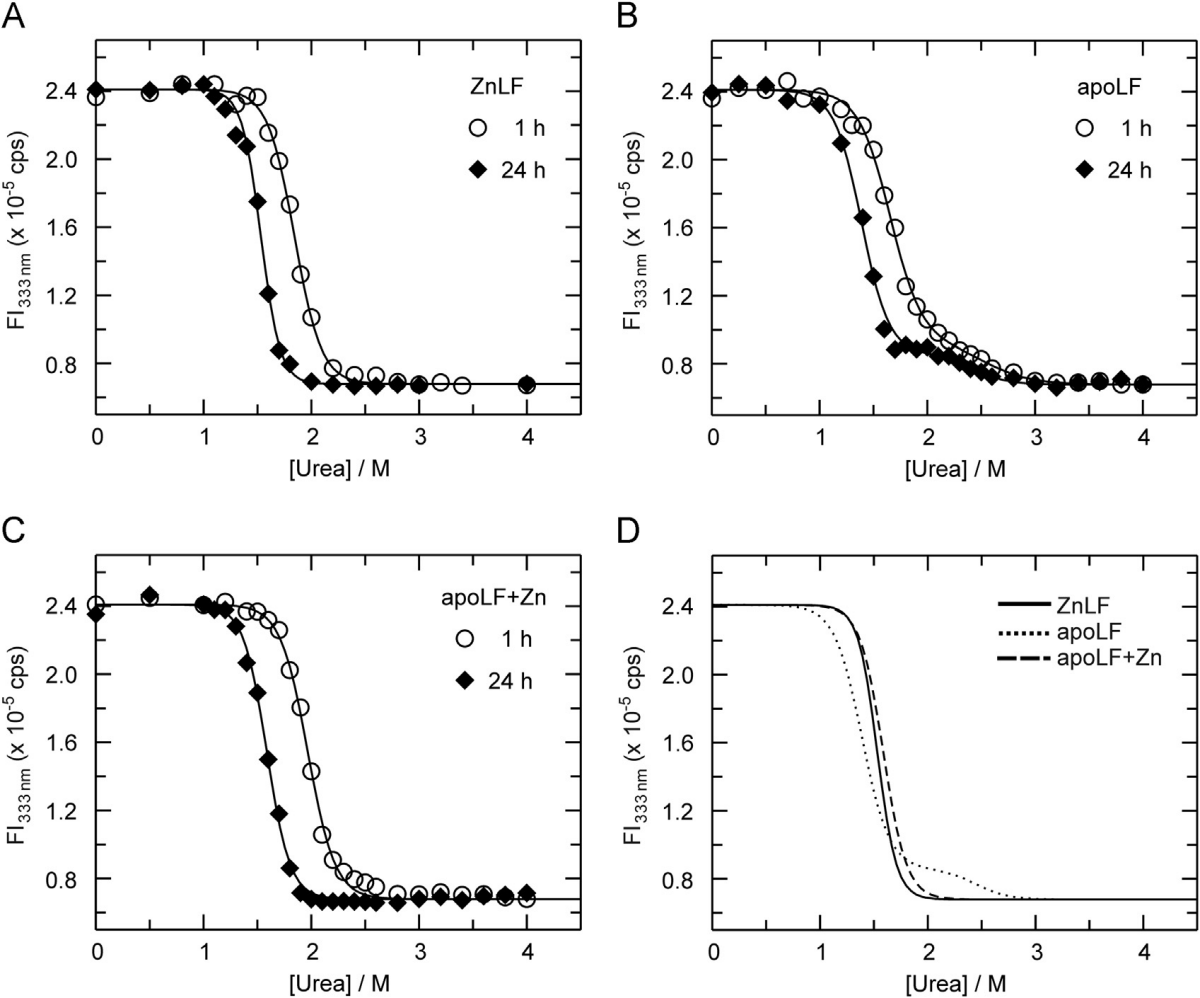
Unfolding profiles for ZnLF, apoLF and Zn^2+^-supplemented apoLF in the presence of urea. Tryptophan fluorescence spectra of all LF samples (0.5 μM final concentration in Hepes buffer) were recorded at 20 °C following incubation of the protein in the absence and presence of urea at the indicated concentrations for 1 h (open circles) and 24 h (closed diamonds). The fluorescence intensity at 333 nm was obtained from each spectrum, and plotted as a function of the concentration of the denaturant. Lines denote the best fit of the data to either Eq. (1), (2). Panel A: Unfolding of ZnLF. The best fit of the data was obtained with Eq. (1) for both 1 h and 24 h samples. Panel B: Unfolding of apoLF (in the presence of 0.1 mM DPA). The best fit of the data was obtained with Eq. (2) for both 1 h and 24 h samples. Panel C: Unfolding of Zn^2+^-reconstituted apoLF. ApoLF (2 μM) was reconstituted with Zn^2+^ (5 μM) for 1 h before dilution (1:4) of the sample with Hepes buffer containing the desired amount of urea. The best fit of the data was obtained with Eq. (1) for both 1 h and 24 h samples. Panel D: Replot of the best fits for the 24 h samples (at equilibrium) of ZnLF (solid line), apoLF (dotted line), and Zn^2+^-reconstituted apoLF (dashed line).

As shown in Fig. 3A, FI_333_ values obtained for ZnLF incubated with urea for 1 h and 24 h could be fit to Eq. (1) describing a two-state transition with midpoint concentrations ($C_{N\to U}^{mid}$) of 1.89 M and 1.55 M, respectively (see Table 2). It is important to point out in this context that the notation of U (unfolded state) is not meant to imply a fully (globally) unfolded state of LF as such assertion would rely on supplementary data from complementary techniques such as circular dichroism (CD) spectroscopy. Instead, the designation of U should be regarded as a descriptor of the folding state following the final structural transition observable by fluorescence spectroscopy.

**Table 2:** Summary of midpoint concentrations.

	Urea		GdnHCl	
	1 h	24 h	1 h	24 h
ZnLF	1.89 (±0.06)	1.55 (±0.03)	0.68 (±0.06)	0.49 (±0.01)
apoLF	1.65 (±0.04)	1.33 (±0.05)	0.65 (±0.08)	0.51 (±0.01)
	2.45 (±0.08)	2.43 (±0.02)		
apoLF+Zn^2+^	1.97 (±0.01)	1.67 (±0.03)	n.d.	n.d.

n.d., not determined. All values (expressed in mol L^−1^) represent the mean (±1 s.d.) of at least three independent experiments, and were calculated (using Eq. (3)) from the $\Delta G^{0}$ and *m* parameters obtained (for each unfolding profile) by fitting of the recorded fluorescence intensities (FI_333_) to Eqs. (1) and/or (2). While values recorded after 24 h of exposure to urea or GdnHCl denote thermodynamic midpoint concentrations, those obtained after 1 h of incubation represent apparent $C^{mid}$ values in view of the lack of a fully established equilibrium after this period of time. Except for urea-denatured apoLF (after both 1 h and 24 h of exposure), the best fit was obtained using the two-state model (see Eq. (1)), with the shown midpoint concentration representing $C_{N\to U}^{mid}$. In the case of urea-exposed apoLF, the best fit was obtained with Eq. (2) (three-state model), yielding $C_{N\to I}^{mid}$ (first value) and $C_{I\to U}^{mid}$ (second value).

An extension of the period of incubation of ZnLF with urea to 48 h did not significantly change the fluorescence intensities (data not shown), suggesting that an equilibrium was reached after 24 h of exposure of LF to the denaturant. Hence, the fitting parameters obtained from the titration data (*i.e.*, $\Delta G^{0}$ and *m*) reflect true thermodynamic (equilibrium) values only for the samples which had been incubated in the presence of denaturant for 24 h (see Table 3). Nonetheless, the data obtained for samples incubated with urea for only 1 h, albeit not at equilibrium, could be fit to the equilibrium equations to obtain an apparent midpoint concentration for the observed transition (see Table 2).

**Table 3:** Equilibrium thermodynamic parameters of urea- and GdnHCl-mediated LF unfolding.

Parameters	Urea			GdnHCl	
	ZnLF	ApoLF	ApoLF+Zn^2+^	ZnLF	ApoLF
**2 State model**					
$\Delta G_{N\to U}^{0}$ (kcal mol^−1^)	10.1 (±0.4)	–	9.6 (±1.1)	7.8 (±0.7)	5.1 (±0.5)
$m_{N\to U}$ (kcal mol^−1^ M^−1^)	6.6 (±0.3)	–	5.7 (±0.8)	15.9 (±1.4)	10.0 (±1.4)

**3 State model**					
$\Delta G_{N\to I}^{0}$ (kcal mol^−1^)	–	6.0 (±0.6)	–	–	–
$m_{N\to I}$ (kcal mol^−1^ M^−1^)	–	4.4 (±0.3)	–	–	–

The parameters $\Delta G^{0}$ and *m* were obtained by fitting the recorded fluorescence intensities (FI_333_) after 24 h of incubation of the LF preparation indicated in the table with urea or GdnHCl to Eq. (1) (two-state model) or Eq. (2) (three-state model). Values represent the mean (±1 s.d.) of three independent experiments, and were calculated by averaging the parameters from each of the three unfolding profiles. The thermodynamic parameters describing the I→U transition (for urea-exposed apoLF only) have been omitted since the standard errors for these fitting parameters were too large (40–50%) to allow for their reliable estimation.

In the case of apoLF, the unfolding profiles (for both 1 h and 24 h periods of incubation) were best fit using the three-state mechanism (Fig. 3B). It is important to note that while the standard errors for the fitting parameters describing the N→I transition (*i.e.*,$\;\Delta G_{N\to I}^{0}$ and $m_{N\to I}$) were small (typically 5% of the parameter values), those of the I→U transition (*i.e.*, $\Delta G_{I\to U}^{0}$ and $m_{I\to U}$) were large (between 40% and 50%), hence, preventing a reliable estimation of the latter thermodynamic parameters. Nonetheless, the distinct shoulder between 2.0 M and 2.5 M urea in the titration profiles (see Fig. 3B) was observed in all three independent titrations performed yielding almost identical $C_{I\to U}^{mid}$ values (see Table 2). The possibility of the shoulder arising from small quantities of ZnLF or reconstituted apoLF is remote since all measurements with apoLF were performed in the presence of DPA. Thus, these results suggest that apoLF unfolds *via* an intermediate state, whose fluorescence signature is similar (yet not identical) to that of the unfolded state. It is interesting to note that fitting the recorded data to a 2-state model (albeit resulting in a poor fit around 2.0 M urea) did yield $\Delta G_{N\to U}^{0}$ and $m_{N\to U}$ values very similar to those obtained for the fit to the 3-state equation for the N→I transition (data not shown), suggesting that the main unfolding transition for apoLF (*i.e.*, N→I) is identical to that of the N→U transition observed for ZnLF.

As shown in Table 2, the midpoint concentration of the N→I transition was found to decrease upon extending the exposure of the protein to urea (from 1 h to 24 h) by 0.32 M, a value similar to that noted for the diminution of the $C_{N\to U}^{mid}$ value for ZnLF (0.34 M). Furthermore, the midpoint concentrations decreased by 0.22–0.24 M with respect to those observed for ZnLF, a finding not surprising in view of the previously documented (slight) stabilization of LF׳s fold in the presence of Zn^2+^ [29], and consistent with the decrease of the Gibbs free energy of unfolding from 10.1 kcal mol^−1^ to 6.0 kcal mol^−1^ (Table 3).

The titration following supplementation of apoLF with Zn^2+^ (see Fig. 3C) revealed the absence of an intermediate state after both 1 h and 24 h of exposure to urea. Indeed, the titration profiles are very similar to those recorded with ZnLF (see Fig. 3D). It is interesting to note that while the $C_{N\to U}^{mid}$ value for the 1 h titration was essentially identical to that obtained for ZnLF, the $C_{N\to U}^{mid}$ value after 24 h of exposure to urea was slightly higher than that observed for ZnLF (see Table 2). Nonetheless, as Table 3 demonstrates, both $\Delta G_{N\to U}^{0}$ and $m_{N\to U}$ values were virtually identical to those determined for ZnLF.

**Fig. 4:**
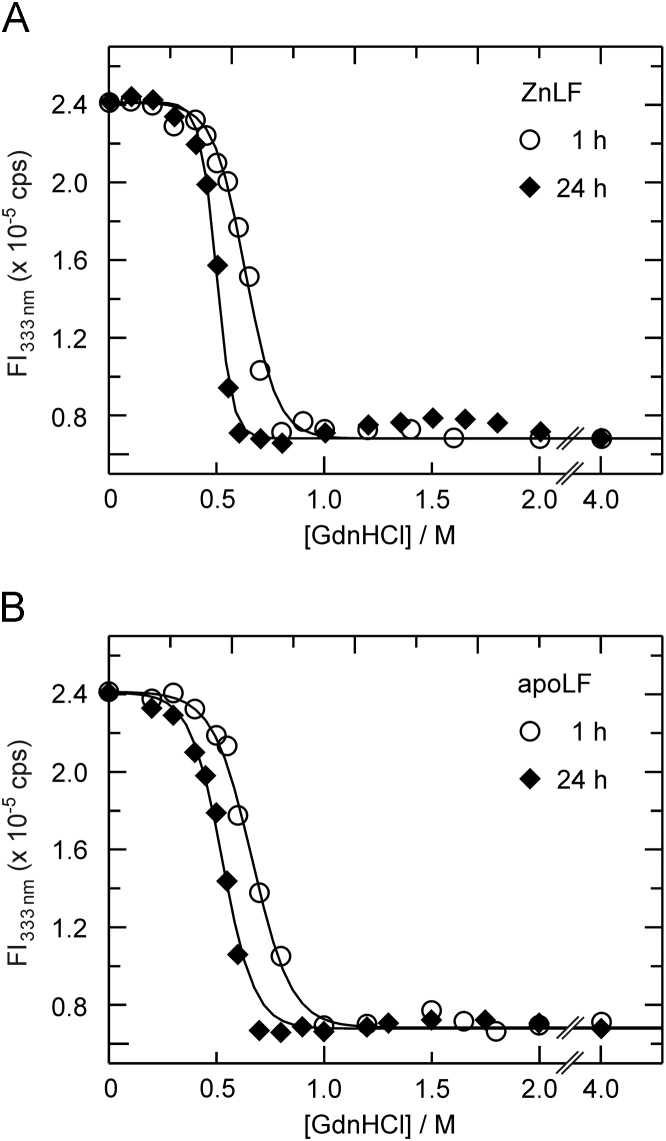
Unfolding profiles for ZnLF (A) and apoLF (B) in the presence of GdnHCl. Fluorescence spectra of ZnLF and apoLF (each 0.5 μM) were recorded at 20 °C following incubation of the protein with GdnHCl for 1 h (open circles) and 24 h (closed diamonds), and the fluorescence intensities (at 333 nm) were plotted as a function of the concentration of the denaturant. Lines denote the best fit of the data to Eq. (1).

The unfolding profiles recorded with GdnHCl could consistently be fit to the two-state model (Eq. (1)) for both the 1 h and 24 h denaturant-exposed ZnLF and apoLF samples (Fig. 4). As Table 2 demonstrates, the results obtained with ZnLF and apoLF parallel those recorded with urea with respect to the decrease in the midpoint concentrations (from 0.68 M to 0.49 M for ZnLF, and from 0.65 M to 0.51 M for apoLF) upon extension of the period of incubation with GdnHCl from 1 h to 24 h. It is interesting to note that, unlike the observations recorded in the case of urea, demetallation of ZnLF did not result in a significant alteration of the $C_{N\to U}^{mid}$ values (see Table 2). However, the Gibbs free energy of unfolding of ZnLF (in the absence of GdnHCl) was found to be higher (by 2.7 kcal mol^−1^) than that recorded for the apoprotein, an observation in accordance with that obtained with urea, and indicative of the enzyme׳s increased stability in the holoprotein form (see Table 3). It is noteworthy that the $\Delta G_{N\to U}^{0}$ values for both ZnLF and apoLF were slightly lower (by 15–22%) than those established from the urea unfolding profiles (see Table 3), although the magnitude of this parameter should in principle be independent of the nature of the denaturant. The values for the $m_{N\to U}$ parameters for both ZnLF and apoLF were significantly larger (by a factor of 2.3–2.4) than those recorded with urea (see Table 3), a finding in accordance with GdnHCl being a much stronger chaotrope than the non-ionic denaturant [30].

### 3.3 Metal release

**Fig. 5:**
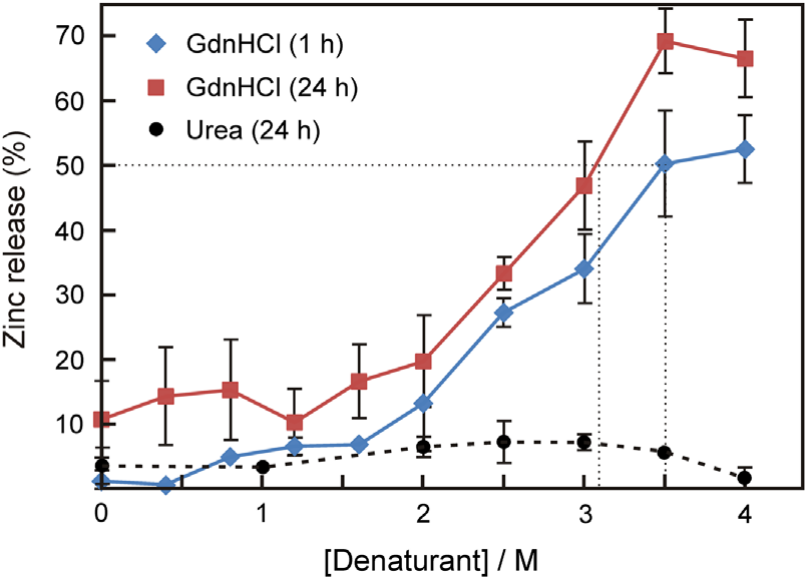
Influence of denaturants on the dissociation of Zn^2+^ from LF. LF (5 μM) in Hepes buffer (50 mM, pH 7.4) was incubated in the absence and presence of GdnHCl or urea at the concentrations indicated in the figure for 1 h (blue diamonds for GdnHCl) and 24 h (red squares for GdnHCl; black circles for urea) at room temperature prior to recovery of released Zn^2+^ by Amicon filtration. The concentration of Zn^2+^ in the filtrate was quantified spectrophotometrically (at 500 nm) with the aid of PAR. Values shown are based on the release of all LF-bound Zn^2+^ ions (*i.e.*, 5 μM) taken as 100%, and depict the mean (±1 s.d.) of three independent experiments. The dotted lines depict the concentrations of GdnHCl at which the midpoints of Zn^2+^ release (50% value) were achieved.

To assess whether the aforementioned structural transitions correlate with an increased lability of LF׳s Zn^2+^ ion, the release of the metal ion from the protein was monitored as a function of the concentration of denaturants by exposing ZnLF to GdnHCl or urea for 1 and 24 h prior to the recovery of any dissociated Zn^2+^ by Amicon filtration, and quantification of the metal ion with PAR. As shown in Fig. 5, LF was found to be capable of retaining its Zn^2+^ ion following a 1 h exposure of the protein to GdnHCl up to concentrations of 1.6 M with respect to the denaturant. At higher concentrations, the degree of Zn^2+^ dissociation progressively increased with the midpoint of metal release (50% value) being observed at a concentration of 3.5 M GdnHCl. In addition, an extension of the time of denaturant exposure of the protein to 24 h yielded (only) a slightly higher degree of Zn^2+^ release (typically about 10±5%) at each concentration of GdnHCl investigated, hence, shifting the midpoint of Zn^2+^ dissociation to a slightly lower concentration (3.1 M). It is interesting to note that while the release of Zn^2+^ was clearly observable with GdnHCl serving as the denaturant, urea was found to be incapable of facilitating the dissociation of the metal ion from the protein (see Fig. 5). Furthermore, the level of Zn^2+^ release from LF was observed to be <10% even at a concentration of urea as high as 6 M, regardless of the duration of exposure (1 h or 24 h) to the denaturant (data not shown). Collectively, these results suggest that LF is capable of retaining its metal ion in a denatured state as evidenced by the significant discrepancies between the midpoint concentrations determined by tryptophan fluorescence spectroscopy (see Table 2) and those obtained from metal release studies.

### 3.4 Accessibility of Zn^2+^ to chelation

**Fig. 6:**
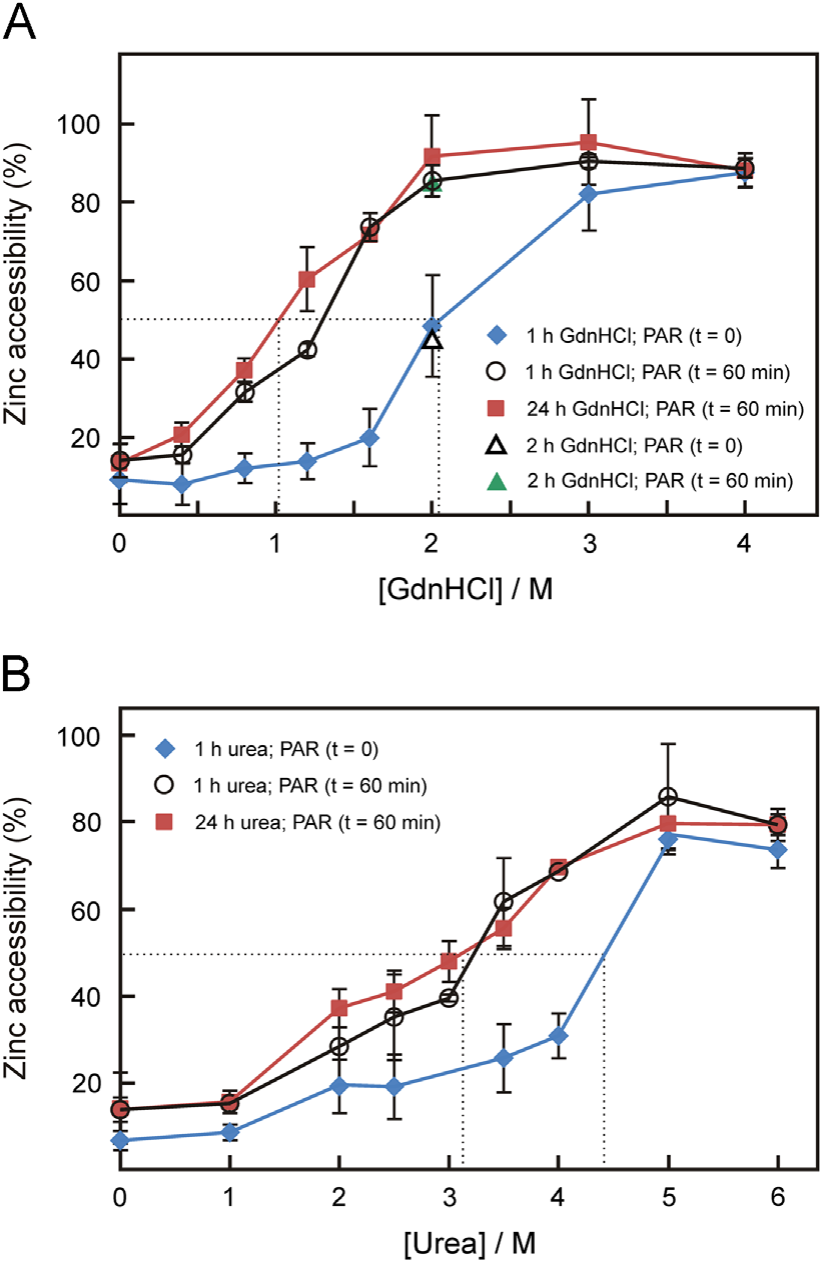
Effect of denaturants on the accessibility of Zn^2+^ to chelation by PAR. LF (5 μM) in Hepes buffer (50 mM, pH 7.4) was exposed to GdnHCl (panel A) or urea (panel B) at the concentrations indicated in the figure for 1 h and 24 h at room temperature prior to the addition of PAR (50 μM final concentration). The progress of the reaction of Zn^2+^ with the chelator was followed spectrophotometrically at 500 nm for 60 min. Full complexation of LF׳s Zn^2+^ ion (*i.e.*, 5 μM given the zinc content of 1.0 Zn^2+^ per LF molecule) denotes an accessibility of 100%. Values shown represent the mean (±1 s.d.) of three independent experiments. The dotted lines represent the concentrations of denaturants at which the midpoints of Zn^2+^ accessibility (50% value) were reached. Panel A: LF was exposed to GdnHCl for 1 h prior to the addition of PAR. The immediate degree of chelation (directly following the addition of PAR; *t*=0) is shown as blue diamonds, whereas that after 60 min of exposure to PAR is depicted as open circles. The Zn^2+^ accessibility of LF exposed to GdnHCl for 24 h before the addition of PAR is shown as red squares. The trace corresponding to the reaction with the chelator for 1 h is shown only since it is identical to that recorded immediately after the addition of PAR. The degree of Zn^2+^ chelation following incubation of LF in the presence of 2.0 M GdnHCl for 2 h is depicted as an open and a closed triangle for the *t*=0 and *t*=60 min exposure times (to PAR), respectively. Panel B: LF was exposed to urea for 1 h prior to the addition of PAR. The immediate degree of chelation (directly following the addition of PAR; *t*=0) is shown as blue diamonds, whereas that after 60 min of exposure to PAR is depicted as open circles. The Zn^2+^ accessibility of LF exposed to urea for 24 h before the addition of PAR is shown as red squares.

In view of the propensity of LF to retain its Zn^2+^ ion at high denaturant concentrations, it was of interest to determine if chaotrope-induced structural changes (observable by fluorescence spectroscopy), although not facilitating the dissociation of the metal ion, might perturb the active site of the enzyme to allow the chelator PAR access to its Zn^2+^ ion. As shown in Fig. 6A, the addition of PAR to LF in the absence of denaturant (1 h incubation in buffer) resulted in the immediate binding of ~10% of Zn^2+^. Even after 1 h of exposure to PAR, no significant increase in the degree of chelation was observed. Since the reaction of PAR with free (unbound) Zn^2+^ in the medium is essentially complete within a few hundred milliseconds (see Fig. S2), these results are indicative of the presence of a small amount of free Zn^2+^ in the sample. Following exposure of LF to 0.8 M GdnHCl for 1 h and subsequent addition of PAR, the immediate level of chelation (*t*=0) was essentially identical to that observed in the absence of the denaturant (~10%). However, after 1 h of incubation with PAR, approximately 30% Zn^2+^ was found to be accessible to chelation. The maximum discrepancy between immediate chelation and that recorded after 1 h of PAR exposure was observed at 1.6 and 2.0 M GdnHCl (see Fig. 6A). At the latter concentration, the midpoint of Zn^2+^ accessibility was observed in view of 50% of the metal ion being captured immediately following the addition of PAR. To ensure that the increase in Zn^2+^ chelation upon incubation with PAR for 1 h was not due to the extension of the exposure to GdnHCl from 1 to 2 h, the Zn^2+^ accessibility after 2 h of incubation with 2.0 M GdnHCl was also monitored. As shown in Fig. 6A, the immediate chelation of Zn^2+^ upon addition of PAR was identical to that of LF treated with GdnHCl for only 1 h. In addition, the degree of Zn^2+^ chelation after 1 h of reaction with PAR was identical to that observed with the corresponding 1 h denaturant-exposed LF sample. This result suggests that the extension of the time of exposure to GdnHCl during the incubation with PAR does not contribute to the recorded increase in Zn^2+^ chelation (from ~50% to ~85%). At 4.0 M GdnHCl, virtually all Zn^2+^ was found to be accessible to chelation irregardless of the time of exposure to PAR.

A more thorough analysis of the time course of the reaction of PAR with LF׳s Zn^2+^ ion revealed the rate of the reaction to be dependent on the denaturant concentration and on time (see Fig. S3). At a GdnHCl concentration ≥2.0 M, the reaction was essentially complete within 5 min, whereas at lower concentrations of the denaturant, significant changes in the level of Zn^2+^ complexation over the course of the first 30 min were observable. No significant alterations in the degree of Zn^2+^ chelation were recorded after 60 min. It is interesting to note that when LF was incubated for 24 h with GdnHCl prior to the addition of PAR, the degree of Zn^2+^ chelation directly following supplementation with the chelator was identical to that observed after 1 h, revealing this process to be time-independent. Indeed, the level of Zn^2+^ accessibility after 24 h of treatment with GdnHCl was found to be very similar to that recorded for LF incubated for 1 h with the denaturant followed by an additional 60 min of exposure to PAR (see Fig. 6A), an observation indicative of an established equilibrium.

While the midpoint of Zn^2+^ accessibility following the incubation of LF with GdnHCl for 24 h was found to be 1.0 M, maximal accessibility was observed at concentrations ≥2.0 M (see Fig. 6A). The latter result is in stark contrast to those obtained from metal release studies where a concentration of>3.0 M of GdnHCl was required to release (only) 50% of LF׳s Zn^2+^ ion. Hence, the degree of the reaction of PAR with the protein׳s metal ion is not due to chelation of any released (free) Zn^2+^, but rather a consequence of the chelator gaining access to the active site metal ion.

In the case of urea, the dependence of the Zn^2+^ accessibility on the concentration of this denaturant (see Fig. 6B) was found to be similar to that noted for GdnHCl in view of the degree of immediate chelation (*t*=0) following exposure of LF to urea for 1 h being significantly smaller than that recorded after 60 min of PAR treatment. Furthermore, the level of Zn^2+^ accessibility observed after incubation of LF with urea for 24 h was similar to that monitored for the 1 h denaturant-exposed protein samples 60 min after supplementation with PAR (see Fig. 6B). The midpoints of Zn^2+^ accessibility were found to be at 4.4 M and 3.1 M for the enzyme incubated in the presence of urea for 1 h and 24 h, respectively. In light of the observation of less than 10% Zn^2+^ being released from LF in the presence of urea (see Fig. 5), these results suggest that the propensity of PAR to scavenge the enzyme׳s metal ion is likely due to its ability to access the active site (and not a consequence of the capture of free Zn^2+^ ions), a finding in accordance with that documented for GdnHCl serving as the denaturant.

## 4 Discussion

A variety of observables can be exploited to assess changes in the structure of proteins as a consequence of perturbations induced by alterations in the pH value, temperature or by the addition of chemical denaturants [31], [32]. In the current study, the influence of two chaotropes, urea and GdnHCl, on the catalytic competence, structural integrity (as monitorered by tryptophan fluorescence spectroscopy) and metal status of LF at neutral pH was investigated in a comparative fashion. Regarding the effect of GdnHCl (and other salts) on the activity of LF, the data presented in this report is consistent with the ionic denaturant inhibiting the enzyme at relatively low concentrations due to its inherent ionic strength (see Table 1), and not due to an induction of protein unfolding. Indeed, an inhibitory effect mediated by NaCl on LF function has previously been reported at a similar ionic strength [33].

As outlined previously, LF harbors five tryptophan residues amenable to investigation by intrinsic tryptophan fluorescence spectroscopy (see Fig. S1). As expected, the treatment of LF with urea and GdnHCl resulted in a redshift of the maximum emission wavelength (from 333 nm to 347 nm) with concomitant quenching of the fluorescence intensity, a phenomenon well established to arise from the exposure of tryptophan residues to a more polar environment [31], [34].

At least in the case of urea, demetallation of ZnLF led to the anticipated diminution in the $C^{mid}$ values. Indeed, in a previous study using urea as the denaturant, the midpoint concentrations (determined based on a two-state mechanism) for ZnLF and apoLF were found to be 2.01 M and 1.64 M, respectively [29]. This observation is similar to that reported herein for the proteins exposed to urea for 1 h (1.89 M and 1.65 M for ZnLF and apoLF, respectively). It is important to note, however, that the time of incubation was not disclosed in the previous report, hence, making a direct comparison to the results reported herein somewhat difficult. In the case of GdnHCl, a change of the $C^{mid}$ values upon demetallation of LF was not observed. However, the analysis of the thermodynamic parameters of GdnHCl-mediated unfolding of ZnLF and apoLF revealed a significant diminution of the $\Delta G_{N\to U}^{0}$ value (by 2.7 kcal mol^−1^ M^−1^) upon demetallation of the protein, an observation which clearly attests to the increased stability of the holoprotein, and which is mirrored in the results documented for urea-exposed LF (see Table 3). Furthermore, studies on the C-terminal core protease domain of LF have shown that although ZnLF and apoLF have essentially the same overall fold, the metal-deficient form is characterized by a slightly lower *α*-helical content, and that binding of the metal ion increases the robustness of the active site [35], [36]. The propensity of Zn^2+^ to induce conformational changes in the catalytic center has also been demonstrated recently for MtfA, a protein whose active site architecture is closely related to that of LF [37].

In addition to the $\Delta G^{0}$ values, the denaturant-dependence of the Gibbs free energy of unfolding (*m* value) was determined. The magnitude of *m* depends on denaturation-induced changes of the solvent-accessible surface area (ΔSASA) of a protein, which can be estimated from the number of amino acid residues [30]. For LF (776 amino acid residues), a ΔSASA value of ~70,000 Å^2^ can be calculated, which translates into *m* values of approximately 16.3 and 8.1 kcal mol^−1^ M^−1^ for GdnHCl and urea, respectively, values which are in general agreement with those obtained for ZnLF in the current study (see Table 3). The *m* values documented for apoLF, although expected to be similar (if not identical) to those obtained for the holoprotein, were found to be smaller for both urea and GdnHCl. It is pertinent to note, however, that the change from a two-state to a three-state mechanism is expected to diminish the *m* value [30], [38], a feature which might explain the lower (than anticipated) *m* value in the case of urea. In this connection it is interesting to note that urea-mediated unfolding of apoLF gave rise to an intermediate state, which was not observed in any of the other titration profiles (including those recorded with GdnHCl). In view of the previously documented slight structural differences between ZnLF and apoLF [35], [36], and the larger Gibbs free energy for the former protein, it is not inconceivable that the unfolding pathways differ between the two protein forms in such a way as to facilitate the detection of the intermediate state with the apoprotein. Since such notion, however, fails to provide an explanation as to why an intermediate state is observable with urea but not with GdnHCl, a few comments regarding the similarities and differences between the two denaturants with respect to the mechanism(s) of unfolding are warranted.

It is well established that the primary facilitator of denaturation appears to be the stabilization of the unfolded protein conformation(s) through solvation of peptide backbone as well as apolar surfaces [39]. Although the intricate details regarding the mechanism of protein (un)folding are still under investigation, recent studies have provided a clearer picture as to how urea and GdnHCl promote these processes. For instance, denaturation by both chaotropes appears to follow a two-state mechanism in which the denaturant interacts first with the protein surface, effectively displacing water molecules from its first hydration shell (and thus forming a dry molten globular state) prior to solvation and disruption of the hydrophobic protein core [40], [41]. In the case of urea, the van der Waals or dispersion forces between the denaturant and the protein backbone or side chains are stronger than those with water, a feature which promotes the access of urea to the protein core [40]. Furthermore, the direct interaction with urea is strongly facilitated by its ability to form hydrogen bonds with the NH moiety of peptide groups [40], [42], [43], thus stabilizing the more open, denatured state. In addition to the significance of urea-backbone interactions, a recent study has shown that an essential contributor to the stability of the urea-denatured state is the more favorable solvation of hydrophobic residues (mainly though dispersion rather than electrostatic forces) [44]. In contrast to urea, GdnHCl does not form hydrogen bonds to peptide groups, but is rather capable of interacting with itself and with planar, non-polar groups in proteins [41], [42]. Hence, GdnHCl most likely disrupts the hydrophobic core of proteins by inserting its flat, hydrophobic surface between non-polar groups of proteins [39], [41]. As such, the observation of an intermediate state for apoLF in urea and its apparent absence in GdnHCl might be a consequence of both denaturants displaying different types of molecular interactions with LF. Hence, it is not inconceivable that only urea is capable of stabilizing the intermediate state encountered with apoLF.

The current investigations on the influence of denaturants on the fold of LF were complemented by studies on the metal release and accessibility using PAR, a chromophoric chelator which has been widely employed to assess the metal content of metalloproteins [45], [46], [47], [48]. In addition, PAR has been utilized to study the copper and zinc release kinetics of Cu,Zn superoxide dismutase (SOD) in the presence of denaturants [49]. In that study, the change in the absorbance of PAR upon exposure of SOD to GdnHCl was interpreted to arise from the denaturant-induced release of the metal ions from the protein. However, as the current study demonstrates, changes following incubation of a metalloprotein in the presence of a denaturant and PAR do not necessarily coincide with the actual metal release as evidenced by the very different midpoints observed in the accessibility and metal release studies using both GdnHCl and urea (see Fig. 5, Fig. 6). Thus, the monitoring of the accessibility of Zn^2+^ in zinc-dependent metalloproteins by the methodology described in this study might serve as an additional parameter to assess denaturant-dependent structural changes in these proteins.

It is apparent from the current study that PAR reacts instantaneously with accessible LF-bound Zn^2+^, and that the chelator is capable of accelerating a structural transition accompanying LF unfolding which is not observable by fluorescence spectroscopy. For instance, following incubation of LF in the presence of urea at a concentration of 3.1 M for 1 h, the immediate degree of chelation was found to be 20%. After 24 h of exposure to the denaturant, Zn^2+^ chelation increased to 50%, and thus to a level identical to that observed for the 1 h-exposed sample treated with PAR for just 60 min (see Fig. 6B). In the case of urea, the results presented here are consistent with the active site Zn^2+^ ion in both the native and denatured states (as observed by tryptophan fluorescence) being inaccessible to PAR since the equilibrium midpoint concentration for the Zn^2+^ accessibility of LF (3.1 M; see Fig. 6B) is twice as high as that recorded for the N→U transition obtained from the unfolding profile of the protein (1.55 M; see Table 2). Indeed, such two-fold difference between the midpoints of accessibility and unfolding was also observed for GdnHCl (see Table 2 and Fig. 6A).

The Zn^2+^-coordinating amino acid residues in LF are located on two distinct α-helices (His686 and His690 on the 4α4 helix, and Glu735 on the 4α7 helix [14]). In view of the narrowness of the PA channel, it has been proposed that structural arrangements as large as an individual α-helix can be guided through the pore [16], [17]. Hence, it is highly unlikely that the coordination environment of the Zn^2+^ ion can be maintained during the translocation process. However, whether the metal ion is lost during this event or whether it is co-transported into the cytosol by being capable of remaining bound to, for instance, the two His residues located on the 4α4 helix, is currently unknown. It is pertinent to note that the fate of LF׳s Zn^2+^ ion during PA-mediated protein transport is not a trivial matter. Although LF has been shown previously to bind Zn^2+^ tightly with picomolar affinity [22], and, if translocated in the apoform, should thus be capable of reinserting the metal ion from cytosolic Zn^2+^ pools [50], [51], a recent study has revealed the combination of PA and apoLF to be non-cytotoxic in macrophage cell-killing assays [23]. This phenomenon has been interpreted to arise from as of yet unidentified structural perturbations in apoLF which prevent the protein from being translocated [23]. Whether this observation is related to the emergence of the intermediate state during the urea-mediated unfolding of apoLF remains to be established. Furthermore, the innate resistance of ZnLF to release its Zn^2+^ ion even when exposed to high denaturant concentrations might be an indicator of the protein-Zn^2+^ complex being the primary species translocated through the PA pore.

In summary, the current report demonstrates that denaturants are capable of impairing LF function prior to the onset of protein unfolding. Furthermore, the enzyme is capable of shielding and retaining its active site Zn^2+^ ion in an (at least partially) unfolded state, a feature indicative of a high degree of metal binding strength. In addition, the Zn^2+^ ion becomes amenable to reaction with PAR (presumably *via* the formation of an enzyme:Zn^2+^:chelator ternary complex) at much lower denaturant concentrations than those required to release the metal ion from the protein. Finally, this study reveals the usefulness of PAR as a probe of metalloprotein structure by being capable of assessing two (as shown here, independent) parameters; the point at which metal ions are accessible to chelation, and the point at which they are released from the protein as a consequence of denaturant exposure. Studies are currently underway to probe the effect of pH on LF׳s fold and metal accessibility/release with a view to more closely mimic the acidic endosomal conditions encountered *in vivo*. In addition, further investigations into the global unfolding of LF (in both its apo- and holo-forms) by complementary techniques such as CD spectroscopy will be required to determine whether structural transitions not observable by fluorescence spectroscopy are facilitating Zn^2+^ ion accessibility and release.

## Appendix B Supplementary materials

Tarnsparency document

Tarnsparency document

Tarnsparency document

Tarnsparency document

## Footnotes

Appendix A Supplementary data associated with this article can be found in the online version at 10.1016/j.bbrep.2015.03.004.
