# Supplementary material for: Influence of chemical denaturants on the activity, fold and zinc status of anthrax lethal factor
Source: Biochem Biophys Rep. 2015 Mar 24;1:68–77. doi: 10.1016/j.bbrep.2015.03.004 (PMC5668564; doi:10.1016/j.bbrep.2015.03.004)
Supplement: Supplementary file 1 — Supplementary material [file mmc1.pdf]

Supplementary Material

Influence of chemical denaturants on the activity, fold and zinc status of anthrax lethal factor

Suet Y. Lo, Crystal E. Säbel, Jonathan P. J. Mapletoft, Stefan Siemann

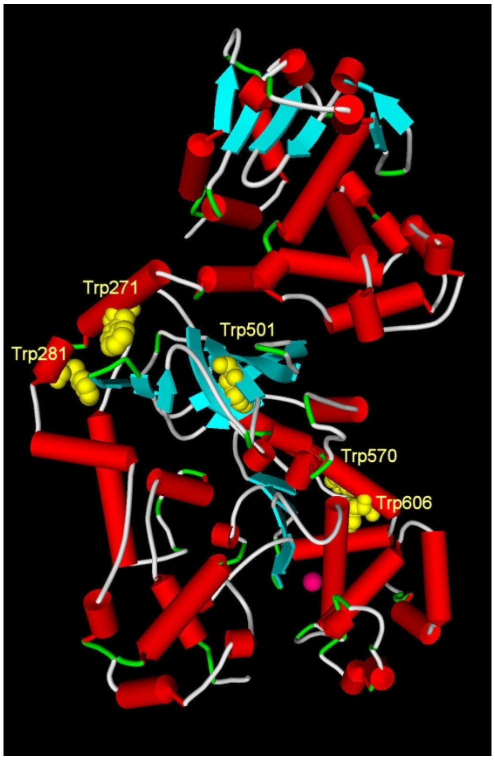

Fig. S1. Location of the five tryptophan residues within the crystal structure of LF. The Trp residues are shown in yellow. The active site Zn^{2+} ion is depicted in magenta. All Trp residues except for Trp271 are deeply buried, and hence essentially solvent inaccessible. Trp271 is semi-buried with a percent solvent accessibility of ~20%. The image was generated with Discovery Studio 3.5 (Accelrys, San Diego, CA) using coordinates deposited under the pdb entry 1J7N [1].

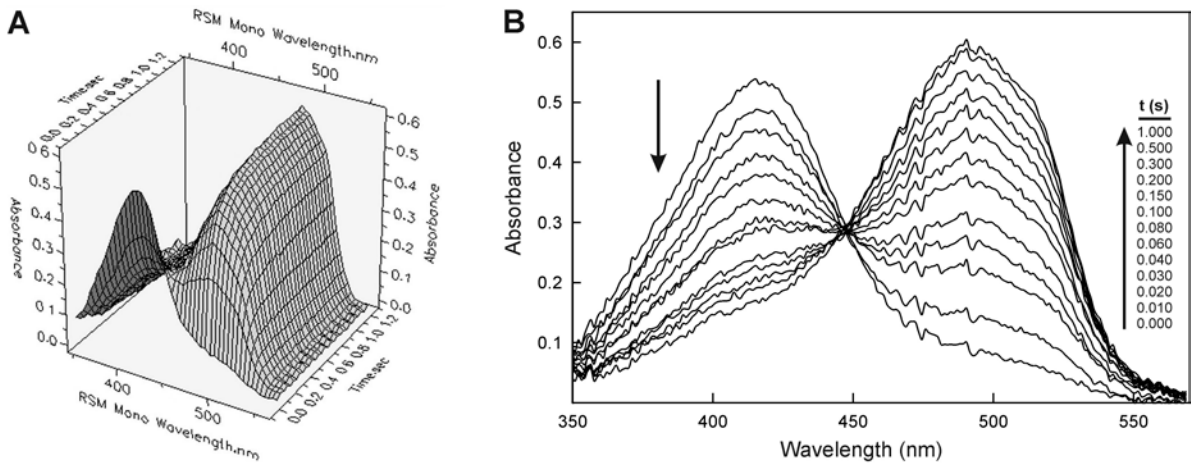

Fig. S2. Stopped-flow spectrophotometric analysis of the reaction of PAR with Zn^{2+}. Rapid-scanning spectra of the complexation of Zn^{2+} by PAR in Hepes buffer (50 mM, pH 7.4) were recorded from 350 nm to 570 nm at room temperature using an OLIS RSM 1000 spectrophotometer (Bogart, GA) equipped with a USA two-syringe stopped-flow device and a cylindrical 20 mm path length cell. A total of 1200 spectra (at 1 ms intervals) were collected. Panel A: 3D plot of the reaction of PAR and Zn^{2+} at final concentrations of 10 μM and 17 μM, respectively. Panel B: 2D representation of the data for the time intervals indicated in the figure. The spectra of free PAR and the PAR:Zn^{2+} complex show maximal absorbance at 416 and 492 nm, respectively, wavelengths in accordance with those previously reported in the literature [2]. The $t_{1/2}$ value for the reaction (i.e., the time required to achieve a concentration of 5 μM with respect to both free and complexed PAR) was found to be 51 ms.

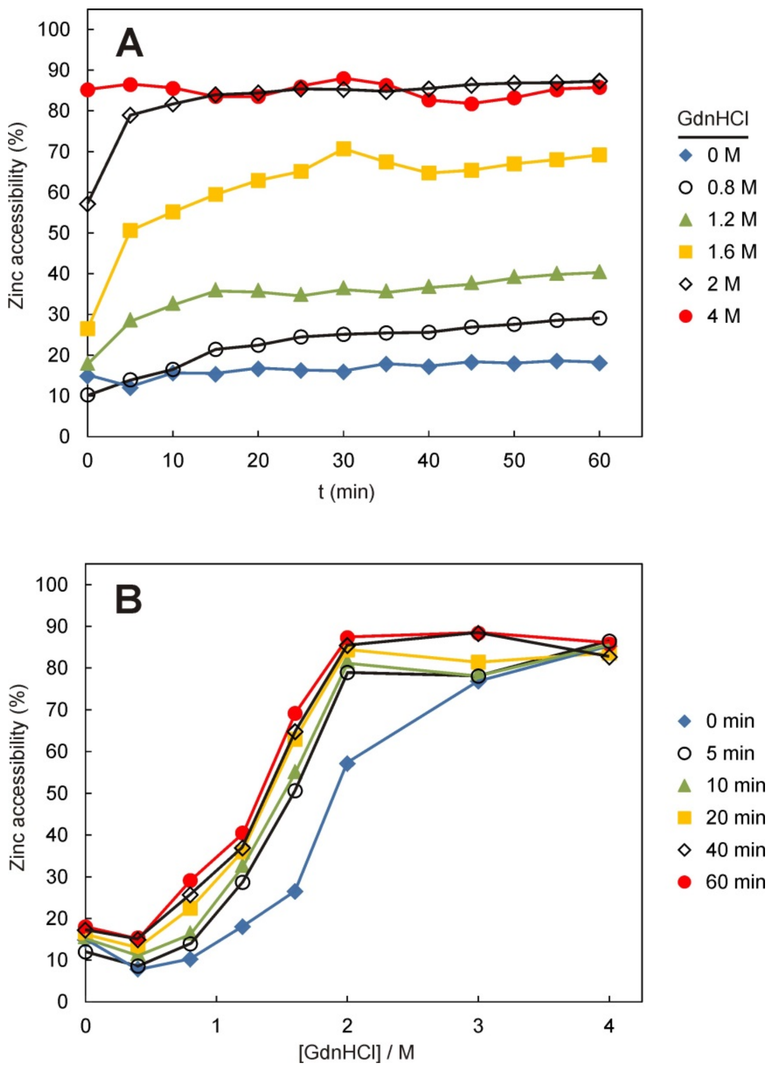

Fig. S3. Influence of time and concentration of GdnHCl on the accessibility of LF's Zn^{2+} ion to chelation by PAR. The degree of chelation of Zn^{2+} was assessed as outlined in the Material and Methods section and as described in the caption of Fig. 6. Panel A: Time-dependence of Zn^{2+} accessibility at the indicated concentrations of GdnHCl. For the sake of clarity, only the data points recorded at 5 min intervals are shown. Panel B: Replot (as a function of the concentration of GdnHCl) of the data shown in panel A with values obtained at concentrations of 0.4 and 3.0 M GdnHCl included.

References:

[1] A.D. Pannifer, T.Y. Wong, R. Schwarzenbacher, M. Renatus, C. Petosa, J. Bienkowska, D.B. Lacy, R.J. Collier, S. Park, S.H. Leppla, P. Hanna, R.C. Liddington, Nature 414 (2001) 229-233.

[2] C.E. Säbel, J.L. Shepherd, S. Siemann, Anal. Biochem. 391 (2009) 74-76.
